# Pregnant outcomes of critically ill pregnant patients with pulmonary hypertension: A multicenter retrospective study

**DOI:** 10.3389/fcvm.2022.872833

**Published:** 2022-09-07

**Authors:** Lin Zhang, Guoqiang Qie, Xiaoyu Yin, Hongyan Zhao, Fusen Zhang, Tao Wang, Mei Meng, Jing Sha, Yufeng Chu

**Affiliations:** ^1^Department of Critical Care Medicine, Shandong Provincial Hospital Affiliated to Shandong First Medical University, Jinan, China; ^2^Department of Critical Care Medicine, The Second Hospital, Cheeloo College of Medicine, Shandong University, Jinan, China; ^3^Department of Intensive Care Unit, Taian Central Hospital, Taian, China; ^4^Department of Intensive Care Unit, Binzhou Medical University Hospital, Binzhou, China; ^5^Department of Critical Care Medicine, Ruijin Hospital, Shanghai Jiao Tong University School of Medicine, Shanghai, China

**Keywords:** pulmonary hypertension, pregnancy, pregnant outcomes, critical care, China

## Abstract

**Objective:**

To identify the pregnancy outcomes and risk factors of critically ill pulmonary hypertension (PH) patients with intensive care unit (ICU) admission.

**Methods:**

The multicenter, retrospective cohort study was performed on 60,306 parturients from January 2013 to December 2018 in China. Diagnosis of PH was based on the estimation of systolic pulmonary arterial pressure (sPAP) *via* echocardiography. Patients were stratified by sPAP into three groups, mild (30–50 mmHg), moderate (51–70 mmHg), and severe (>70 mmHg). The primary outcome was major adverse cardiovascular events (MACE), defined as a composite of in-hospital death, heart failure, and sustained arrhythmias requiring treatment. The secondary outcome was fetal adverse clinical events (FACE), a composite of fetal/neonatal death, prematurity, small birth weight, and fetal distress.

**Results:**

A total of 181 pregnant patients were enrolled, including 101 patients with mild PH, 31 with moderate PH, and 49 with severe PH. The maternal median age was 32 (27, 35) years and 37% were nulliparous. The MACE occurred in 59 (59/181, 32.6%) women, including in-hospital death in 13 (13/181, 7.2%), heart failure in 53 (53/181, 29.3%), and sustained arrhythmias in 7 (7/181, 3.9%). The incidence of FACE was as high as 66.3% (120/181). Compared with mild and moderate PH patients, patients with severe PH had a significantly higher mortality rate (22.4 vs. 1.51%, *P* < 0.001) and MACE incidence (51.0 vs. 25.8%, *P* = 0.001). Although the incidence of FACE in severe PH was slightly higher than that in mild to moderate PH, there was no significant difference (69.4 vs. 65.1%, *P* = 0.724). PH complicated with left heart disease (OR = 4.365, CI: 1.306–14.591), elevated N-terminal pro-B-type natriuretic peptide (NT-proBNP) level (OR = 1.051, CI:1.015–1.088), and sPAP level estimated by echocardiography (OR = 1.021; CI: 1.003–1.040) were independently associated with MACE in multivariable regression (*P* < 0.05). Increased risk of FACE was noted for PH patients combined with eclampsia/preeclampsia (OR = 6.713; CI: 1.806–24.959).

**Conclusion:**

The incidence of MACE and FACE remained high in critically ill pregnant patients with PH, particularly moderate and severe PH in China. Further studies are warranted to identify subsets of women with PH at lower pregnant risks and seek more effective therapy to improve pregnancy outcomes.

## Introduction

Pulmonary hypertension (PH) is a complex and devastating disease often leading to severe right heart failure and death. Women with PH are usually advised to avoid pregnancy because it is known to be associated with high maternal mortality (even up to 30–56%), as well as an extremely high incidence of fetal adverse clinical events (33–100%) (1–3). In reality, despite adequate counseling, some women with PH still chose to take great risks to get pregnant or continue unplanned pregnancies. In addition, recent data from the real world show that the incidence of pregnancy in women with PH is increasing, which may be due to the improvement in the diagnosis and treatment, so that more female patients with PH could survive to child-bearing age, and even have a near-normal life (4, 5).

Advances in the management of PH and pregnancy have significantly improved the pregnancy outcome of patients with PH. Recently, a series of studies, including a large national contemporary data set in the United States and a retrospective study from China, have reported lower mortality (0.8–6.4%) of PH patients with pregnancy than previously reported (6). Therefore, the individualized risk-based approach may be more appropriate for pregnancy in PH than the most recent guidelines recommendation to avoid pregnancy in all these patients. As most deaths occurred postpartum, the intensive care unit (ICU) has become the main battlefield for rescuing critically ill pregnant women. However, there are limited data on pregnancy outcomes and risk factors of critically ill PH patients admitted to the ICU.

The aim of this study was to investigate maternal and fetal outcomes in PH women with ICU admission. The clinical characteristics, complicated diseases, type of anesthesia, mode of delivery, and medication were evaluated, as well as the risk factors associated with postpartum maternal major adverse cardiovascular events (MACE) and fetal adverse clinical events (FACE).

## Methods

### Study design and participants

**Figure 1:**
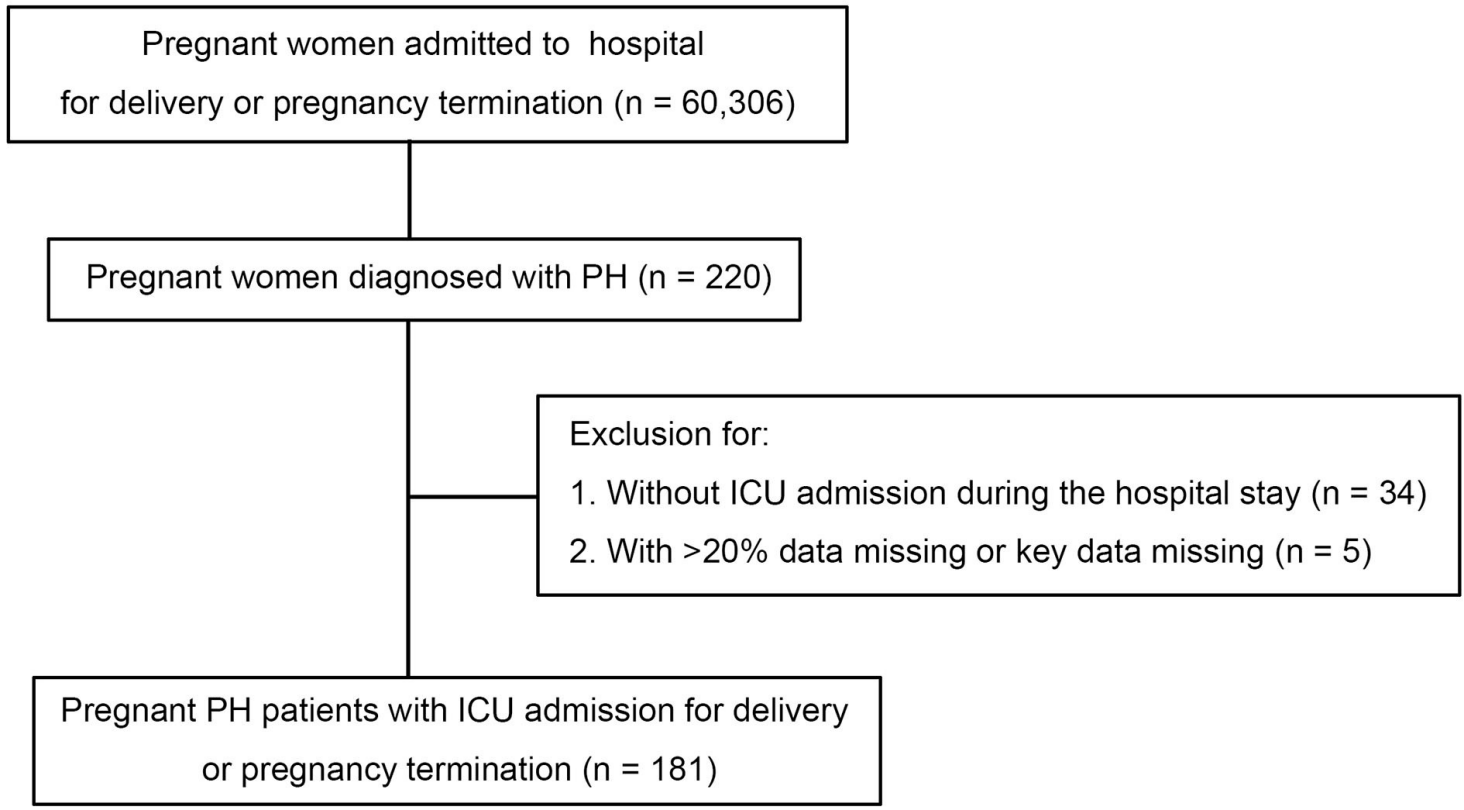
Study population and process of inclusion. ICU, intensive care unit; PH, pulmonary hypertension.

The multicenter retrospective study was conducted at three provincial maternal referral centers, including Shandong Provincial Hospital Affiliated to Shandong First Medical University, Taishan Hospital Affiliated to Shandong First Medical University, and Binzhou Medical University Hospital. All pregnancies including miscarriages, ectopic pregnancies, terminations, and completed pregnancies were enrolled. The pregnant patients with PH consecutively admitted to the ICU from January 1, 2013 to December 31, 2018 were included and analyzed. Patients with more than 20% data missing or key data missing were excluded (see Figure 1).

### Data collection

All data were extracted from the electronic medical system. Two clinical researchers reviewed the electronic medical records and collected the data, then a third researcher determined any differences between the interpretations of the two primary reviewers. For patients with readmission during the study period, data from the first admission were presented. Survival after discharge was obtained by telephone interviews.

### Diagnostic criteria and definitions of clinical parameters

Diagnosis of PH was based on the estimation of systolic pulmonary arterial pressure (sPAP) *via* echocardiography. When patients were admitted, echocardiography was performed by a qualified sonographer as described in the 2015 ESC/ERS guideline (3). Patients were stratified by sPAP into three groups, mild (30–50 mmHg), moderate (51–70 mmHg), and severe (>70 mmHg) (2). In addition, patients were divided into four groups according to the related disease: iPH, patients with an initial diagnosis of idiopathic PH before this admission and without any known cause of PH; CHD-PH, patients with a previous history of congenital heart disease; LHD-PH, patients with a history of left ventricular systolic dysfunction caused by valvular disease, cardiomyopathy, or acquired left heart outflow tract obstruction, but not congenital structural abnormality; oPH, PH caused by other diseases. For more definitions see Supplementary material 1.

### Outcomes

The primary outcome was MACE, defined as a composite of in-hospital death, heart failure, and sustained arrhythmias requiring treatment (4, 7). The secondary outcome was FACE, a composite of fetal/neonatal death, prematurity, small birth weight, and fetal distress (8).

### Ethical approval

Given the retrospective design of the study, which was based on data extracted from the electronic medical system, written informed consent was not required. All the procedures performed in this study were in accordance with the 1964 Helsinki declaration and its later amendments or comparable ethical standards. This study was registered in the Chinese Clinical Trial Registry and was approved by the Ethics Committee of Shandong Provincial Hospital (Registration number: ChiCTR1900020624).

### Statistical analysis

The distributions of variables were examined with Kolmogorov–Smirnov tests. Continuous data were expressed as mean ± standard deviation or medians (interquartile range, IQR) as appropriate. Groups were compared by student *t*-tests or ANOVA analysis if they obey a normal distribution. If not, they were compared by Mann-Whitney U-tests or Kruskal-Wallis tests. Categorical data were expressed as numbers (percentages) and were compared by the chi-square test or Fisher's exact tests. Cut-off points were presented *via* receiver operating characteristic (ROC) curves. Risk factors for MACE and FACE were determined with univariate logistic regression and multivariate logistic regression. Odds ratios (OR) with 95% confidence intervals (CI) and the corresponding *P*-values were calculated for each risk factor. Risk factors for in-hospital maternal death were determined with univariate cox regression and multivariate cox regression. Hazard ratios (HRs) with 95% confidence intervals (CIs) and the corresponding *P*-values were calculated for each risk factor. Kaplan–Meier survival analysis was used to describe the relative risk of death and the log-rank test was used to compare the survival probability of each group. *P*-values were considered statistically significant if <0.05 (two-sided test). The variables that had >5% of values missing were excluded. Another missing data imputation was done using the Expectation-Maximization method. All statistical analysis was performed using SPSS 25.0 (IBM Corporation, Armonk, New York, USA).

## Results

### Patient characteristics

From January 2013 to December 2018, a total of 60,306 deliveries were performed at the three provincial maternal referral centers and 220 (3.65 per 1,000 births) had PH. Of patients with PH, 186 (84.5%) were admitted to ICU. Five patients were excluded for missing data. In the final analysis, 181 patients were included in this study. Among all the pregnant patients with PH, 49 (27.1%) cases had an sPAP above 70 mmHg, 31 (17.1%) cases had an sPAP between 50 and 70 mmHg, and 101 (55.8%) cases had an sPAP below 50 mmHg. The maternal median age was 32 (27, 35) years and 37% were nulliparous. In more than 65% of patients, the PH diagnosis was made before pregnancy. However, no patients received targeted therapy for PH before or during pregnancy. The median gestational age on admission was 35 (32, 37) weeks, and there were no differences in mild, moderate, and severe PH groups. On admission, the sign of heart failure was presented in more than one-third of the pregnant women. Overall, women with severe PH were associated with higher NYHA cardiac classification compared to those with mild and moderate PH (*P* < 0.001). It was found that iPH was present in 33 (18.2%) patients, CHD-PH in 83 (45.9%), LHD-PH in 15 (8.3%), and oPH in 50 (27.6%) patients. Further baseline characteristics are presented in Table 1.

**Table 1:** Baseline characteristics of pregnant women based on PH severity diagnosed *via* echocardiography.

**Variable**	**Total** **(*n* = 181)**	**Mild PH** **(*n* = 101)**	**Moderate PH (*n* = 31)**	**Severe PH** **(*n* = 49)**	***p*-value**
**Age, years [M, (Q1, Q3)]**	32 (27, 35)	29 (33, 37)	26 (31, 37)	24 (30, 34)	0.014
≤25 y (*n*, %)	34 (18.8)	13 (12.9)	4 (12.9)	17 (34.7)	
25-35 y (*n*, %)	92 (50.9)	51 (50.5)	17 (54.8)	24 (49.0)	
≥35 y (*n*, %)	55 (30.4)	37 (36.6)	10 (32.3)	8 (16.3)	
Nulliparous (n, %)	67 (37.0)	32 (31.7)	11 (35.5)	24 (49.0)	0.081
BMI, kg/m^2^	26.8 (23.8, 30.9)	28.2 (25.0, 32.6)	26.5 (23.8, 29.6)	24.8 (22.4, 27.7)	0.000
**Diagnosis made (** ***n*** **, %)**					0.096
Before pregnancy	120 (66.3)	60 (58.6)	24 (77.4)	36 (73.5)	
During pregnancy	61 (33.7)	41 (40.6)	7 (22.6)	13 (26.5)	
**Treatment (** ***n*** **, %)**					
Targeted PH therapy	0 (0)	0 (0)	0 (0)	0 (0)	NS
Anticoagulation	2 (1.1)	1 (1.0)	1 (3.2)	0 (0)	0.452
Gestational week on admission, weeks [M, (Q1, Q3)]	35 (32, 37)	36 (32, 38)	36 (33, 38)	34 (31, 37)	0.112
Saturation, % [M, (Q1, Q3)]	100 (98, 100)	100 (98, 100)	100 (97, 100)	100 (98, 100)	0.981
MAP, mmHg [M, (Q1, Q3)]	92.0 (82.8, 108.8)	102.3 (82.8, 117.7)	88.7 (80.3, 99.3)	89.0 (79.8, 100.3)	0.014
Hemoglobin, g/L [M, (Q1, Q3)]	114.0 (102.0, 124.0)	113.0 (101.0, 119.0)	111.0 (102.0, 127.0)	116.0 (105.0, 134.05)	0.001
Hematocrit, % [M, (Q1, Q3)]	35.2 (32.3, 37.7)	35.0 (31.8, 36.9)	33.8 (32.2, 38.0)	35.9 (32.9, 40.8)	<0.001
Platelets, × 10^9^ /L [M, (Q1, Q3]	193 (146, 230)	194.0 (147.0, 227.0)	218.0 (175.0, 281.0)	178.0 (115.0, 219.0)	0.031
D-dimer, mg/L [M, (Q1, Q3)]	1.69 (1.02, 3.38)	2.2 (1.3, 3.7)	1.4 (0.9, 1.9)	1.3 (0.6, 4.0)	0.006
CRP, mg/L [M, (Q1, Q3)]	4.38 (2.33, 13.2)	4.5 (2.7, 13.7)	3.8 (1.7, 11.2)	4.9 (2.2, 15.6)	0.473
NT-proBNP, pg/ml [M, (Q1, Q3)]	490.6 (185.0, 1368.0)	424.7 (184.3, 1056.5)	495.4 (58.4, 1505.0)	595.7 (295.1, 2038.0)	0.201
Heart failure (*n*, %)	75 (41.4)	27 (26.7)	17 (54.8)	31 (63.3)	<0.001
**NYHA class (** ***n*** **, %)**					<0.001
I	76 (42.0)	53(52.5)	12 (38.7)	11 (22.4)	
II	55 (30.4)	35 (34.7)	10 (32.3)	10 (20.4)	
III	36 (19.9)	13 (12.9)	4 (12.9)	19 (38.8)	
IV	14 (7.7)	0 (0)	5 (16.1)	9 (18.4)	
APACHE II	3 (2,4)	3 (2,4)	3 (2,4)	3 (2,5)	0.838
SOFA score [M, (Q1, Q3)]	1 (0, 2)	1 (0, 2)	1 (0, 2)	1 (0, 2)	0.576
**Echocardiographic parameters**					
sPAP, mmHg [M, (Q1, Q3)]	49.0 (41.0, 73.0)	42.0 (38.0, 46.0)	58.0 (53.0, 66.0)	91.0 (81.0, 105.0)	<0.001
Diameter of main PA, cm [M, (Q1, Q3)]	2.7 (2.41, 3.17)	2.7 (2.4, 3.0)	3.0 (2.2, 3.2)	3.2 (2.5, 3.7)	0.075
**Complications (** ***n*** **, %)**					
Hypertension	56 (30.9)	44 (43.6)	5 (16.1)	7 (14.3)	<0.001
Pre-eclampsia/eclampsia	49 (27.1)	40 (39.6)	5 (16.1)	4 (8.2)	<0.001
HELLP syndrome	1 (0.6)	1(1.0)	0 (0)	0 (0)	NS
Diabetes mellitus	14 (7.7)	10 (9.9)	1 (3.2)	3 (6.1)	0.594
Autoimmune disease	11 (6.1)	8 (8.9)	0 (0)	3 (6.1)	0.393
Liver damage	5 (2.8)	1 (1.0)	2 (6.5)	2 (4.1)	0.095
Kidney injury	9 (5.0)	5 (5.0)	1 (3.2)	3 (6.1)	0.901
Others	15 (8.3)	10 (9.9)	2 (6.5)	3 (6.1)	0.757
**Related diseases (** ***n*** **, %)**					
iPH	33 (18.2)	23 (22.8)	4 (12.9)	6 (12.2)	0.099
CHD-PH	83(45.9)	34 (33.7)	14 (45.2)	35 (71.4)	<0.001
Eisenmenger syndrome	8 (4.4)	0 (0)	1 (3.2)	7 (14.3)	<0.001
Left-to-right shunts	29 (16.0)	11 (10.9)	4 (12.9)	14 (28.6)	0.075
LHD-PH	15 (8.3)	9 (8.9)	5 (16.1)	1 (2.0)	0.075
oPH	50 (27.6)	35 (34.7)	8 (25.8)	7 (14.3)	0.041
Length of hospital stay, days [M, (Q1, Q3)]	7 (5, 9)	7 (5, 9)	7 (5, 9)	7 (5, 10)	0.898
Length of ICU stay, days [M, (Q1, Q3)]	2 (2, 4)	2 (1, 3)	2 (2, 3)	3.5 (2, 5)	<0.001

APACHE II score, Acute Physiology, Age and Chronic Health Evaluation II score; BMI, body mass index; CHD-PH, patients with the previous history of congenital heart disease; CRP, C-reactive protein; HELLP syndrome: hemolysis, elevated liver enzymes, and low platelets syndrome; ICU, intensive care unit; iPH, patients with an initial diagnosis of idiopathic pulmonary hypertension before this admission and without any known cause of pulmonary hypertension; LHD-PH, patients with a history of left ventricular systolic dysfunction but not congenital structural abnormality; M, median; MAP, mean arterial pressure; NT-proBNP, N-terminal pro-B-type natriuretic peptide; NYHA, New York Heart Association; oPH, pulmonary hypertension associated with other disease; PA, pulmonary artery; PH, pulmonary hypertension; Q1, the first quartile; Q3, the third quartile; SOFA score, sequential organ failure assessment score; sPAP, systolic pulmonary arterial pressure estimated via echocardiography.

### Management and clinical characteristics of patients

Management of pregnant patients with PH is presented in Table 2. The median gestational age at delivery was 35 (32, 38) weeks. Cesarean section (CS) was performed in 174 patients (96.1%), of which 37 (20.4%) were emergency operations. Only 7 (3.9%) women delivered virginally. Compared with mild (*n* = 20, 19.8%) and moderated (*n* = 6, 19.4%) PH patients, more women with severe PH (*n* = 24, 49%) received general anesthesia. At ICU admission, compared with mild and moderate PH patients, the patients with sPAP >70 mmHg showed higher sequential organ failure assessment (SOFA) score, Acute Physiology, Age and Chronic Health Evaluation II (APACHE II) score, blood lactate level, D-dimer level, and more PaO_2_/FiO_2_ decrease. Further treatments are presented in Table 2.

**Table 2:** Management of pregnant patients with pulmonary hypertension.

**Variable**	**Total** **(*n* = 181)**	**Mild PH** **(*n* = 101)**	**Moderate PH** **(*n* = 31)**	**Severe PH** **(*n* = 49)**	***p-*value**
Delivery, weeks	35 (32, 38)	36 (32, 38)	37 (34, 38)	34 (31, 37)	0.185
**Mode of delivery (** ***n*** **, %)**					
Vaginal	7(3.9)	2 (2.0)	1 (3.2)	4 (8.2)	0.020
Cesarean section	174 (96.1)	99 (98.0)	30 (96.8)	45 (91.8)	0.021
Emergency cesarean section	37 (20.4)	16 (15.8)	6 (19.4)	15 (30.6)	0.232
**Anesthesia (** ***n*** **, %)**					
General	51 (28.2)	20 (19.8)	6 (19.4)	24 (49.0)	0.002
Epidural	19 (10.5)	10 (9.9)	5 (16.1)	4 (8.2)	0.538
Spinal	1 (0.6)	1 (1.0)	0 (0)	0 (0)	1.000
Epidural+Spinal	108 (59.7)	70(69.3)	19 (61.3)	19 (38.8)	0.003
**On ICU admission**					
APACHE II score, [M, (Q1, Q3)]	5 (3, 7)	4 (3, 6)	5 (3, 7)	6 (4, 8)	0.001
SOFA score [M, (Q1, Q3)]	1 (1, 2)	1 (1, 2)	1 (1, 3)	2 (1, 4.0)	0.023
Lactate, mmol/L [M, (Q1, Q3)]	1.2 (1.0, 1.7)	1.1(0.9, 1.4)	1.3 (0.9, 2.0)	1.5 (1.2, 2.8)	0.000
NT-proBNP, pg/ml [M, (Q1, Q3)]	451.2 (201.7, 1299.5)	440.7 (196.6, 1057.8)	730.1 (208.5, 1457.8)	548.7 (219.75, 1669.5)	0.532
cTnI, ng/ml [M, (Q1, Q3)]	10.7 (6.93, 22.1)	10.8 (6.9, 19.2)	9.1 (6.3, 16.1)	12.6 (5.9, 39.3)	0.489
**PaO2/FiO2**					<0.001
≤100 (*n*, %)	13 (7.2)	1 (1.0)	1 (3.2)	11 (22.4)	
100–300 (*n*, %)	109 (60.2)	66 (65.3)	17 (54.8)	26 (53.1)	
>300 (*n*, %)	59 (32.6)	34 (33.7)	13 (41.9)	12 (24.5)	
Hemoglobin, g/L [M, (Q1, Q3)]	106.0 (94.0, 120.0)	113.0 (101.0, 119.0)	111.0 (102.0, 127.0)	116.0 (105.0, 135.0)	0.113
Hematocrit, % [M, (Q1, Q3)]	32.8 (29.4, 35.8)	35.0 (31.8, 36.9)	33.8 (32.2, 38.0)	35.9 (32.9, 40.8)	0.003
Platelets, × 10^9^ /L [M, (Q1, Q3)]	177.0 (128.0, 228.0)	194.0 (147.0, 227.0)	218.0 (175.0, 281.0)	178.0 (115.0, 219.0)	0.222
D-dimer, mg/L [M, (Q1, Q3)]	3.71 (2.18, 7.72)	4.3 (2.7, 10.9)	2.6 (1.8, 4.1)	3.2 (2.0, 8.3)	0.033
CRP, mg/L [M, (Q1, Q3)]	5.63 (1.83, 21.7)	4.1 (1.5, 18.3)	16.4 (2.1, 35.5)	10.1 (3.2, 26.3)	0.173
Acute kidney injury (*n*, %)		6 (5.9)	1 (3.2)	3 (6.1)	1.000
**Treatment (*****n**,* **%)**					
Inotropic agents	16 (8.8)	5 (5.0)	3 (9.7)	8 (16.3)	0.062
Vasoconstrictors	21 (11.6)	3 (3.0)	4 (12.9)	13 (26.5)	<0.001
Anticoagulation	121 (66.9)	72 (71.3)	22 (71.0)	27 (55.1)	0.099
Targeted PAH therapy	2 (1.1)	0 (0)	1 (3.2)	1 (2.0)	0.195
CRRT	9 (5.0)	3 (3.0)	1 (3.2)	5 (10.2)	0.187
ECMO	1 (0.5)	0 (0)	0 (0)	1 (2.0)	0.440
**Fluid balance, ml [M, (Q1, Q3)]**					
1st 24 h	−375 (−1321, 210)	−651 (−1686, 105)	−493 (−1058, 231)	−231 (−814, 233)	0.084
2nd 24 h	−386(−1202, 140)	−421 (−1406, 270)	−250 (−1249, 55)	−386 (−1104, 97.5)	0.965
3rd 24 h	−150 (−735, 480)	−97.5 (−658, 1125)	32.2 (−590, 384.5)	−211 (−777, 269)	0.592

APACHE II score, Acute Physiology, Age and Chronic Health Evaluation II score; cTnI, cardiac troponin I; CRP, C-reactive protein; CRRT, continuous renal replacement therapy; ECMO, extracorporeal membrane oxygenation; ICU, intensive care unit; M, median; NT-proBNP, N-terminal pro-B-type natriuretic peptide; PH, pulmonary hypertension; PaO_2_/FiO_2_, the ratio of arterial oxygen partial pressure to fractional inspired oxygen; Q1, the first quartile; Q3, the third quartile; SOFA score, sequential organ failure assessment score.

### Maternal and fetal outcomes

**Figure 2:**
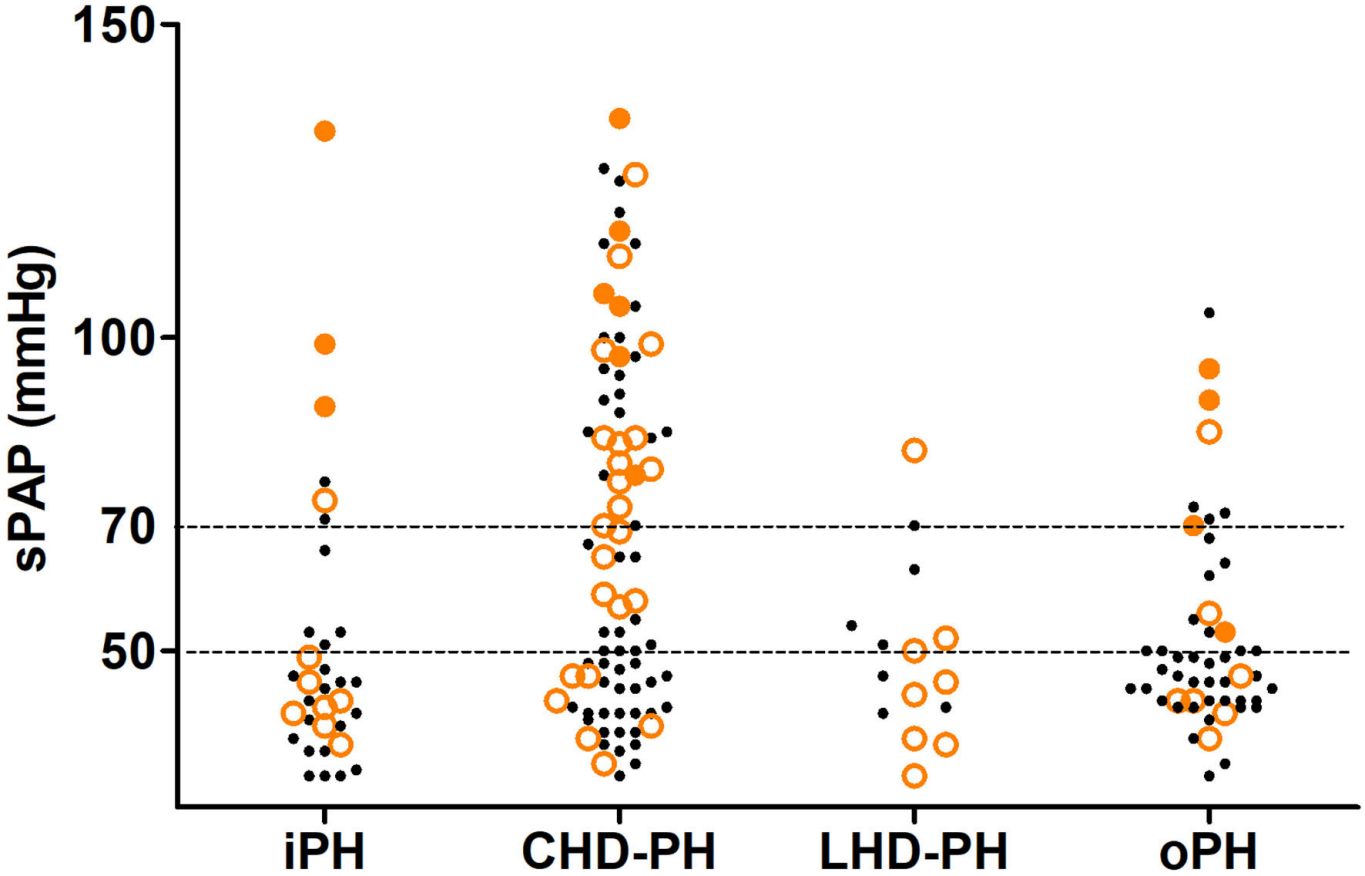
In-hospital outcome of the pregnancy and different severities of pulmonary hypertension in the study population based on the related disease. The small black dots represent the cases without MACE, the lager orange circles represent the cases that experience MACE, and the large orange dots represent the cases that experience in-hospital death. CHD-PH, patients with the previous history of congenital heart disease; iPH, patients with an initial diagnosis of idiopathic pulmonary hypertension before this admission and without any known cause of pulmonary hypertension; LHD-PH, patients with a history of left ventricular systolic dysfunction but not congenital structural abnormality; MACE, major adverse cardiovascular events; oPH, pulmonary hypertension associated with other diseases; sPAP, systolic pulmonary arterial pressure estimated *via* echocardiography.

**Figure 3:**
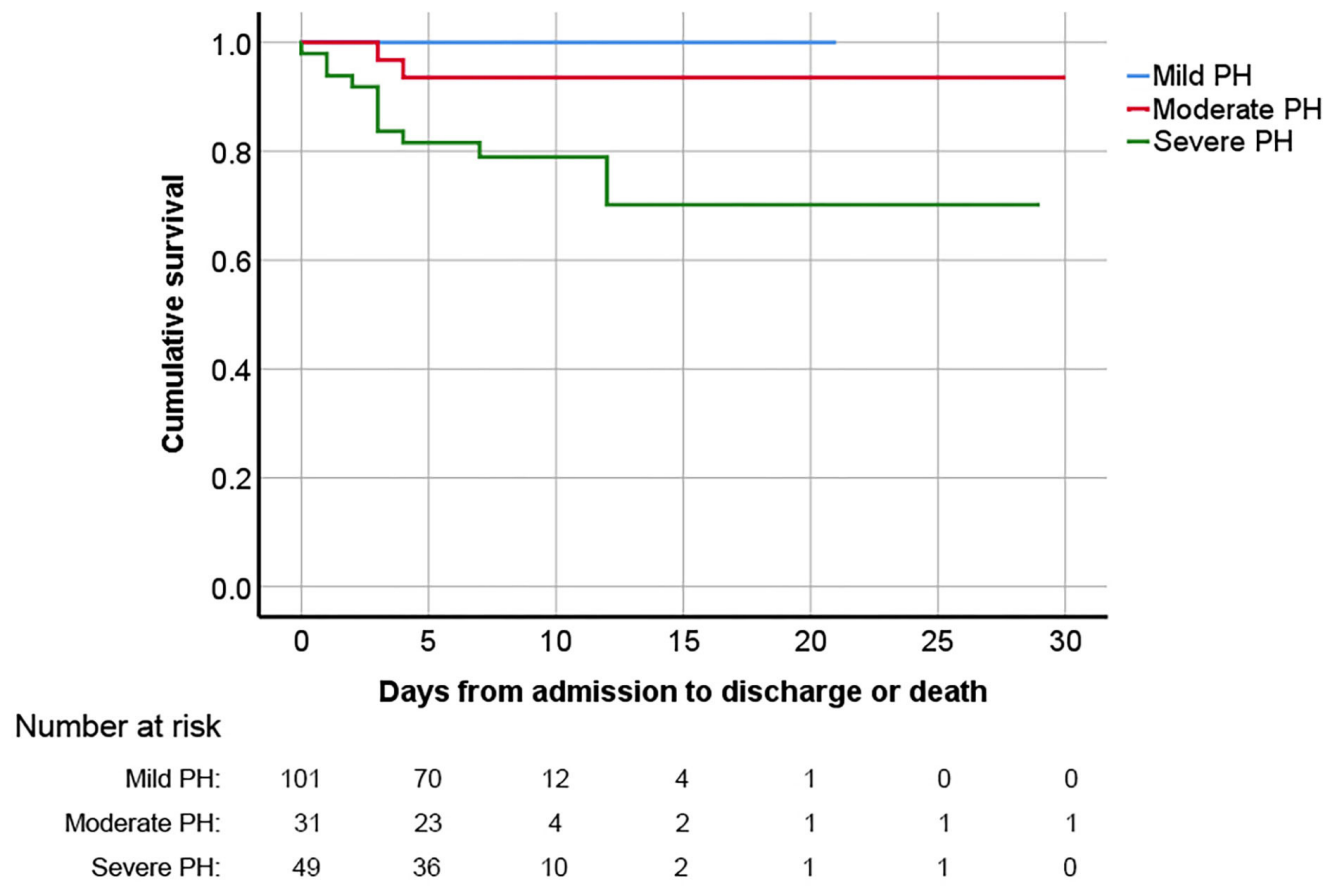
Kaplan–Meier survival analysis curve for pregnant women with PH diagnosed *via* echocardiography based on PH severity. PH, pulmonary hypertension.

The in-hospital outcomes of these pregnant PH women are shown in Table 3. The MACE occurred in 59 (32.6%) women: in-hospital death in 13 (7.2%), heart failure in 53 (29.3%), and sustained arrhythmia in 7 (3.9%). Compared with patients with sPAP ≤70 mmHg, women with sPAP above 70 mmHg experienced significantly higher MACE (51.0 vs. 25.8%, *P* = 0.001). The related diseases and severity distribution of PH parturients who experienced MACE are shown in Figure 2. The in-hospital survival outcomes of these pregnant PH women are shown in Figure 3. Compared with mild and moderate PH patients, patients with severe PH had a significantly higher mortality rate (22.4%, *P* < 0.001). Of all the 13 in-hospital deaths, 1 died of pulmonary hypertension crisis and 12 died of heart failure. There were two deaths in patients with moderate PH. One patient (sPAP = 53 mmHg) died of valvular thrombosis-induced heart failure, which was due to previous aortic valve replacement, postpartum hemorrhage, and anticoagulant discontinuation. The other patient (sPAP = 70 mmHg) died of a sudden cardiac arrest. In addition, most patients died within 1 week postpartum (3 died within 24 h and 9 died within 24 h to 1 week postpartum), and one woman died 12 days after delivery. Moreover, a total of 168 patients were followed up for at least 1 year after discharge. During the follow-up,1 patient died of heart failure 116 days after discharge and one patient died of cerebral hemorrhage 110 days after discharge. Both the two patients had sPAP above 70 mmHg.

**Table 3:** Maternal and fetal/neonatal outcomes of women with PH diagnosed *via* echocardiography.

**Variable**	**Total**	**Mild PH**	**Moderate PH**	**Severe PH**	***p*-value**
**Maternal outcome**					
Death	13 (7.2)	0 (0)	2 (6.5)	11 (22.4)	<0.001
During pregnancy (*n*, %)	0 (0)	0 (0)	0 (0)	0 (0)	1.000
Postpartum (<24 h) (*n*, %)	3 (1.7)	0 (0)	0 (0)	3 (6.1)	0.023
Postpartum (24 h−1 w) (n, %)	9 (5.0)	0 (0)	2 (6.5)	7 (14.3)	<0.001
Postpartum (>1 w, <6 w) (*n*, %)	1 (0.6)	0 (0)	0 (0)	1 (2.0)	0.439
Sustained arrhythmia (*n*, %)	7 (3.9)	1 (1.0)	3 (9.7)	3 (6.1)	0.033
Heart failure (*n*, %)	53 (29.3)	23 (22.8)	8 (25.8)	22 (44.9)	0.008
Thromboembolic event (*n*, %)	3 (1.7)	1 (1.0)	0 (0)	2 (4.1)	0.255
Postpartum hemorrhage (*n*, %)	8 (4.4)	6(5.94)	1 (3.2)	1 (2.0)	0.695
MACE (*n*, %)	59 (32.6)	24 (23.8)	10 (32.2)	25 (51.0)	0.001
**Fetal/neonatal outcome**					
Live births (*n*, %)	155 (85.6)	88 (87.1)	29 (93.5)	38 (77.5)	0.137
Prematurity (birth <37 w) (*n*, %)	108 (59.7)	62 (61.4)	16 (51.6)	30 (61.2)	0.598
Therapeutic abortion	17 (9.4)	8 (7.9)	1 (3.2)	8 (16.3)	0.154
Fetal death (*n*, %)	6 (3.3)	4 (4.0)	0(0)	2 (4.1)	0.727
Pregnancy loss (*n*, %)	23 (12.7)	12 (11.9)	1 (3.2)	10 (20.4)	0.087
Low birth weight (*n*, %)	63 (34.8)	35 (34.7)	10 (32.2)	18 (36.7)	0.928
Fetal distress (*n*, %)	9 (5.0)	6 (5.9)	1 (3.2)	2 (4.1)	1.000
Neonatal malformation (*n*, %)	1 (0.6)	0 (0)	1 (3.2)	0 (0)	0.167
Neonatal death (*n*, %)	3 (1.7)	2 (2.0)	0 (0)	1 (2.0)	1.000
FACE (*n*, %)	120 (66.3)	69 (68.3)	17 (54.8)	34 (69.4)	0.928

FACE, fetal adverse clinical events; MACE, major adverse cardiovascular events; PH, pulmonary hypertension.

The FACE occurred in 120 (66.3%) women, including 17 (9.4%) therapeutic abortion, 6 (3.3%) fetal death, 3 (1.7%) neonatal death, 108 (59.7%) prematurity, 63 (34.8%) small birth weight, and 9 (5.0%) fetal distress. Although the incidence of FACE in patients with severe PH was slightly higher than that in patients with mild to moderate PH, there was no significant difference (69.4 vs. 65.1%, *P* = 0.724). Further maternal and fetal/neonatal outcomes are presented in Table 3.

### Risk factors for maternal and fetal outcomes

The result of univariable logistic and cox regression is presented in Supplementary material 2. The result of multivariable logistic and cox regression is shown in Tables 4, 5. MACE was more likely to occur in patients with higher sPAP, higher NT-BNP level, higher lactate level, higher cTnI level, and lower PaO_2_/FiO_2_. Under the age of 25, heart failure on admission, acute kidney injury, LHD-PH, emergency cesarean section, and higher APECHE II and SOFA scores were considered possible risk factors for MACE. Patients in whom the FACE occurred had higher BMI, higher NT-proBNP, higher SOFA score, and lower PaO_2_/FiO_2_. Hypertension, eclampsia/preeclampsia, CHD-PH, and general anesthesia were all considered possible risk factors for FACE. According to the multivariable logistic regression analysis, the development of MACE was associated with the complication of left heart disease (OR = 4.365, CI:1.306–14.591), and elevated NT-proBNP level (OR = 1.051, CI:1.015–1.088) and sPAP level estimated by echocardiography (OR = 1.021; CI: 1.003–1.040). Combined with eclampsia/preeclampsia was considered to be a risk factor for FACE (OR = 6.713; CI: 1.806, 24.959). Delivery week >32 weeks was proved to be protective in FACE (OR = 0.056, CI: 0.007–0.444). According to the cox regression, in-hospital maternal death was much more likely to happen in patients with higher sPAP estimated by echocardiography (HR = 1.056; CI: 1.029–1.084), higher NT-proBNP level on admission (HR = 1.011; CI: 1.001–1.020) and higher lactate level on ICU admission (HR = 1.135; CI: 1.020–1.263). As presented in the ROC curves (see Supplementary material 3), the cut-off of sPAP, NT-proBNP on admission, and lactate level on ICU admission was 70 mmHg, 1,000 pg/ml, and 2.85 mmol/L, respectively.

**Table 4:** Multivariable logistic regression of risk factors for MACE and FACE in pregnant patients with PH diagnosed *via* echocardiography.

**Variable**	**Multivariable logistic regression**	
	**OR (95%CI)**	***p-*value**
**MACE (*****n*** **=** **59, 32.6%)**		
LDH-PH	4.365 (1.306, 14.591)	0.017
NT-proBNP	1.051 (1.015, 1.088)	0.005
sPAP	1.021 (1.003, 1.040)	0.025
**FACE (*****n*** **=** **120, 66.3%)**		
Delivery week greater than 32wks	0.056 (0.007, 0.444)	0.006
Combined with eclampsia/preeclampsia	6.713 (1.806, 24.959)	0.004

FACE, fetal adverse clinical events; LHD-PH, patients with a history of left ventricular systolic dysfunction but not congenital structural abnormality; MACE, major adverse cardiovascular events; NT-proBNP, N-terminal pro-B-type natriuretic peptide; sPAP, systolic pulmonary arterial pressure.

**Table 5:** Multivariable cox regression of risk factors for maternal mortality in pregnant patients with PH.

**Variable**	**Multivariable cox regression**	
	**HR (95%CI)**	***p*-value**
**In hospital maternal death (*****n*** **=** **13, 7.2%)**		
sPAP	1.056 (1.029–1.084)	<0.001
NT-proBNP on admission	1.011 (1.001–1.020)	0.031
Lactate level on ICU admission	1.135 (1.020–1.263)	0.020

ICU, intensive care unit; NT-proBNP, N-terminal pro-B-type natriuretic peptide; sPAP, systolic pulmonary arterial pressure.

## Discussion

In this retrospective multicenter study, we analyzed clinical characters, complications, treatments, as well as maternal and fetal/neonatal outcomes of 181 pregnant PH women in the past 6 years. The incidence of MACE (32.6%) and FACE (66.3%) remained extremely high despite the continuous progress of treatment in recent years. Pregnant women with LHD-PH, high NT-proBNP levels, and higher sPAP levels were risk factors for the development of MACE. In addition, high sPAP (≥70 mmHg) estimated *via* echocardiography elevated NT-proBNP (≥1,000 pg/ml) on admission, as well as high lactate level (≥2.85 mmol/L) on ICU admission had a good predictive value for maternal death. Combined with eclampsia/preeclampsia was independently associated with FACE development.

Consistent with previous studies, patients with LHD-PH experienced the most MACE. There were 53.3% (8 out of 15) LHD-PH patients who experienced MACE, which was much higher than the other three groups (34.9% in CHD-PH patients, 33.3% in iPH patients, and 22.0% in oPH patients). In a European registry of 1,321 pregnant women with cardiac disease, patients with cardiomyopathy, commonly combined with left ventricular dysfunction, had the most adverse cardiac events (9). Different from the systolic and diastolic capacity of right ventricular injury caused by long-lasting increased afterload, LHD-PH often occurred as a consequence of a passive filling pressure of the left heart, which was driven by left ventricular dysfunction and left atrium compliance loss (10, 11). After delivery, uterine contraction and increased blood volume would furtherly aggravate the condition. However, despite the relatively high incidence of adverse events, all of the LHD-PH women survived, and most of them underwent a successful delivery, which was consistent with previous reports (6, 12). The possible reason was that most of the underlying heart diseases were generally monitored and well-controlled before pregnancy, and most of the LHD-PH patients were in the mild to moderate PH group.

Pregnancy with PH has a high risk of death. Compared with the maternal mortality rate (18.3/100,000) in China in 2018, the mortality rate of pregnant patients complicated with PH increased by nearly 400 times in the present study. Similar to our study, higher mortality was also reported in other studies but showed a decreasing trend (1, 13, 14). A meta-analysis including 20 studies and 589 cases reported a pooled maternal mortality of 11.5%. The data from a European registry of 151 pregnant PH women showed a mortality of 3.3% within 1 week postpartum, and 4.6% during 6-month postpartum follow-up (2). In another study of 1,519 patients using the U.S. database data, the in-hospital maternal mortality was 0.8% (6, 15). The authors attributed this relatively lower maternal mortality to collaborative multidisciplinary team management, planned termination of pregnancy before 28 weeks, and preference for spinal or epidural anesthesia. Therapeutic abortion was also found helpful in other researches; however, the mode of delivery and anesthesia remained contentious (16). In this study, we also found that no death occurred in patients who terminated pregnancy before 28 weeks, including patients with severe PH in this study, suggesting that early termination of pregnancy might improve the prognosis of pregnant women with PH, but more real-world studies are warranted.

Although invasive hemodynamic measurements are still the gold standard for PH diagnosis, a detailed echocardiographic assessment of pulmonary artery pressure could help to identify patients at risk of PH. Accumulating evidence suggested a correlation between non-invasive and invasive data, but the reported results were not completely consistent (17, 18). For an individual patient, significant overestimation and underestimation might occur. Recent guidelines have suggested that although echocardiography could not provide a definitive diagnosis, it was of great significance in determining the probability of PH being present (19). In addition, for pregnant women with confirmed or moderate to severe PH, echocardiography might be more suitable for dynamic prediction and follow-up because of its non-invasive and convenient. Consistent with recent research (20), the level of sPAP measured by echocardiography was closely associated with mortality and morbidity of postpartum adverse events in pregnant PH women in our study. Furthermore, the optimal cut-off value of sPAP for predicting mortality was 70 mm Hg, which yielded sensitivity and specificity of 88.9 and 71.1%, respectively.

In this study, we found that pregnant PH women with high levels of NT-proBNP had higher mortality and more cardiac adverse events. NT-proBNP levels have also been included in the multiparametric risk assessment approach for PH outlined in PH guidelines (3, 21). In PH patients, NT-proBNP was secreted by the ventricular myocardium in response to transmural pressure, volume overload as well as hypoxia (22). As demonstrated in a previous study including PH patients both newly diagnosed and receiving long-term treatment, change in NT-proBNP level was correlated to the changes in right ventricular function (23). However, data supporting the use of NT-proBNP risk thresholds in assessing pregnancy risk in PH are limited and inconsistent. Further studies are required to confirm the prospective use of NT-pro BNP in this field.

We noticed a high FACE incidence rate of 66.3% in our study. Compared with a recent meta-analysis that reviewed 589 parturients and 610 pregnancies in 20 studies, there are similar pregnancy loss rate (12.7 vs. 12.6%) and a higher prematurity rate (59.7 vs. 51.7%) in this study (16). Planned early delivery was widely applied to avoid adverse events in our study, which was a possible reason for this high prematurity rate. In our study, PH combined with eclampsia/preeclampsia was associated with FACE. One explanation for poor fetal outcomes of PH patients was limited placental development due to decreased cardiac output (2). Placental disease due to vascular endothelial injury was also recognized as the main cause of fetal restriction and stillbirth in eclampsia/preeclampsia. As reported, women with PH were more likely to experience eclampsia, which was a risk factor for both maternal and fetal adverse events (6, 24). Furthermore, pulmonary artery pressure would significantly increase in the third trimester, and most of the patients with severe PH had NYHA class decline by 1 or 2 (25). Considering maternal and fetal safety, despite the patients' strong desire of continuing pregnancy, most of them were advised to terminate the pregnancy early. The specific influence of PH on placental development remained unclear. Further research on fetal outcomes was still needed.

The major limitation of the study was related to its retrospective nature. In this study, all data were extracted from the electronic medical system, and some of the data were incomplete or unavailable. In addition, the diagnosis of PH was not made with a right heart catheterization. It is inevitable to overestimate or underestimate the value of sPAP in an individual patient leading to misdiagnosis and inappropriate treatment. The interpretation of our data must take into account the limitations of echocardiography in the diagnosis of PH. Thus, all conclusions must be drawn with caution.

In conclusion, maternal mortality remained high despite advanced treatment, and the incidence of MACE and FACE was also very high in this multicenter data set of critically ill pregnant women with PH in China. PH patients with left heart disease, increased sPAP estimated by echocardiography, and elevated NT-proBNP are at high risk of cardiac adverse events and should receive closer medical monitoring. If necessary, planned early delivery should be considered to avoid sudden deterioration of cardiac function. However, further studies are warranted to identify subsets of women with PH at lower pregnant risks and seek more effective therapy to improve pregnancy outcomes.

## Data availability statement

The raw data supporting the conclusions of this article will be made available by the authors, without undue reservation.

## Ethics statement

The studies involving human participants were reviewed and approved by Medical Ethics Committee of Provincial Hospital Affiliated to Shandong University. Written informed consent from the participants' legal guardian/next of kin was not required to participate in this study in accordance with the national legislation and the institutional requirements.

## Author contributions

YC and MM contributed to conception and design of the study. GQ, XY, HZ, FZ, and TW contributed to data acquisition and outcome measure. LZ and YC performed the statistical analysis. LZ wrote the first draft of the manuscript. YC, MM, and JS revised the manuscript critically. All authors contributed to manuscript revision, read, and approved the submitted version.

## Funding

The work was supported by Foster fund of the Second Hospital, Cheeloo College of Medicine, Shandong University (2022YP73), Clinical Medical Science and Technology Innovation Project of Jinan (202134023), and Shandong traditional Chinese Medicine Science and Technology Project (2021M184).

## Conflict of interest

The authors declare that the research was conducted in the absence of any commercial or financial relationships that could be construed as a potential conflict of interest.

## Publisher's note

All claims expressed in this article are solely those of the authors and do not necessarily represent those of their affiliated organizations, or those of the publisher, the editors and the reviewers. Any product that may be evaluated in this article, or claim that may be made by its manufacturer, is not guaranteed or endorsed by the publisher.

## Abbreviations

APACHE II score, Acute Physiology: Age and Chronic Health Evaluation II score
BMI: body mass index
CHD-PH: pulmonary hypertension complicated with congenital heart disease
cTnI: cardiac troponin I
CRP: C-reactive protein
CRRT: continuous renal replacement therapy
ECMO: extracorporeal membrane oxygenation
FACE: fetal adverse clinical events
HELLP syndrome, hemolysis, elevated liver enzymes: and low platelets syndrome
ICU: intensive care unit
iPH: idiopathic pulmonary hypertension
LHD-PH: pulmonary hypertension complicated with left heart disease
M: median
MACE: major adverse cardiovascular events
MAP: mean arterial pressure
NT-proBNP: N-terminal pro-B-type natriuretic peptide
NYHA: New York Heart Association
oPH: pulmonary hypertension associated with other disease
PA: pulmonary artery
PaO_2_/FiO_2_: the ratio of arterial oxygen partial pressure to fractional inspired oxygen
PH: pulmonary hypertension
Q1: the first quartile
Q3: the third quartile
SOFA score: sequential organ failure assessment score
sPAP: systolic pulmonary arterial pressure estimated *via* echocardiography.
