# Supplementary material for: Pregnant outcomes of critically ill pregnant patients with pulmonary hypertension: A multicenter retrospective study
Source: Front Cardiovasc Med. 2022 Sep 7;9:872833. doi: 10.3389/fcvm.2022.872833 (PMC9489930; doi:10.3389/fcvm.2022.872833)
Supplement: Supplementary file 1 [file Data_Sheet_1.docx]

Supplementary Material

**Diagnostic criteria and definitions of clinical parameters**

When patients were admitted, echocardiography was performed by qualified sonographer. As described in 2015 ESC/ERS guideline, the sPAP was estimated based on the tricuspid regurgitation velocity (TRV) and right arterial pressure (RAP). The tricuspid transvalvular pressure was calculated according to the simplified Bernoulli equation based on the TRV. The RAP was estimated based on the diameter of the inferior vena cava (IVC): RAP was estimated as 3 mmHg in most cases, if and IVC diameter >2.1 cm that collapses <50% with a sniff or <20% on quiet inspiration, the RAP would be estimated as 15 mmHg. The sPAP was estimated as the sum of tricuspid transvalvular pressure and RAP, if there was no right ventricle outflow obstruction. The PH diagnosis was established if the sPAP was above 30 mmHg(1). Patients were stratified by sPAP into three groups, mild (30-50 mmHg), moderate (51-70 mmHg), and severe (>70 mmHg). In addition, patients were divided into four groups according to the related disease: idiopathic PH (iPH), PH complicated with congenital heart disease (CHD-PH), PH complicated with left heart disease (LHD-PH), and PH caused by other diseases (oPH)(2).

Body mass index (BMI) was defined as the body mass (kg) divided by the square of body height (m). Heart failure sign was defined as symptoms induced by reduced heart ability, including dyspnea, fatigue and weakness, persistent cough with or without pink blood-tinged phlegm, edema, ascites, rapid or irregular heartbeat, severe hypotension excluding hypovolemic shock as well as any other type of shock. The extent of heart failure was assessed using New York Heart Association Functional Classification (NYHA) system(3). Hypertension was defined as systolic blood pressure ≥140 mmHg and/or diastolic blood pressure ≥90 mmHg which was measured for at least two times in different days(4). Pre-eclampsia/eclampsia was defined as the presence of hypertension and proteinuria or other comorbidities with or without seizure in a previously normotensive pregnant woman after 20th week of gestation(5). HELLP syndrome was defined as presence of hemolysis, elevated liver enzyme and low platelet count in pre-eclampsia women(5). Diabetes mellitus was defined as elevated plasma glucose level resulting from type 1 Diabetes, type 2 Diabetes, and gestational Diabetes. Autoimmune disease was defined as disease caused by malfunctional attack of immune system (exclude type 1 Diabetes). Liver damage was defined as the peak value of alanine aminotransferase above threefold of the upper limit of normal. Acute kidney injury was defined according to the KDIGO clinical practice guideline(6). The Sequential Organ Failure Assessment (SOFA) score and Acute Physiology and Chronic Health Evaluation (APACHE II) score were calculated separately using the worst value of physiological variables within 24 h of presentation. Emergency caesarean section was defined as the caesarean section performed in emergency situation without detailed plan and were not yet fully discussed as a lifesaving procedure. Thromboembolic event was defined as all the thrombosis related disease happened after delivery, including deep venous thrombosis, pulmonary embolism and sinus thrombosis, etc. Arrhythmia was defined as sustained abnormal heart beats with an irregular or abnormal rhythm occurred after delivery, including ventricular fibrillation, ventricular tachycardia, supraventricular tachycardia, atrial fibrillation, etc. Postpartum haemorrhage was defined as blood loss of more than 500 ml with in the 1st 24 hours after delivery. Prematurity was defined as the neonate born before 37 weeks of pregnancy. Therapeutic abortion was defined as the ending of a pregnancy on therapeutic purpose. Pregnancy loss was defined as the death of unborn fetus during pregnancy. Fetal death was defined as the spontaneous intrauterine death of a fetus at any time during pregnancy. Fetal distress was defined as fetal compromise due to inadequate oxygen supply. The characteristic fetal heart rate patterns of fetal distress were late decelerations, variable decelerations, and prolonged bradycardia. Neonatal malformation was defined as structural or functional anomalies that occurred during intrauterine life. Neonatal death was defined as death happened during the 1st 28 days after birth.

1. Galie N, Humbert M, Vachiery JL, Gibbs S, Lang I, Torbicki A, et al. 2015 Esc/Ers Guidelines for the Diagnosis and Treatment of Pulmonary Hypertension: The Joint Task Force for the Diagnosis and Treatment of Pulmonary Hypertension of the European Society of Cardiology (Esc) and the European Respiratory Society (Ers): Endorsed By: Association for European Paediatric and Congenital Cardiology (Aepc), International Society for Heart and Lung Transplantation (Ishlt). *Eur Respir J* (2015) 46(4):903-75. Epub 2015/09/01. doi: 10.1183/13993003.01032-2015.

2. Sliwa K, van Hagen IM, Budts W, Swan L, Sinagra G, Caruana M, et al. Pulmonary Hypertension and Pregnancy Outcomes: Data from the Registry of Pregnancy and Cardiac Disease (Ropac) of the European Society of Cardiology. *Eur J Heart Fail* (2016) 18(9):1119-28. Epub 2016/07/08. doi: 10.1002/ejhf.594.

3. Yancy CW, Jessup M, Bozkurt B, Butler J, Casey DE, Jr., Drazner MH, et al. 2013 Accf/Aha Guideline for the Management of Heart Failure: Executive Summary: A Report of the American College of Cardiology Foundation/American Heart Association Task Force on Practice Guidelines. *Circulation* (2013) 128(16):1810-52. Epub 2013/06/07. doi: 10.1161/CIR.0b013e31829e8807.

4. Unger T, Borghi C, Charchar F, Khan NA, Poulter NR, Prabhakaran D, et al. 2020 International Society of Hypertension Global Hypertension Practice Guidelines. *Hypertension* (2020) 75(6):1334-57. Epub 2020/05/07. doi: 10.1161/HYPERTENSIONAHA.120.15026.

5. Gestational Hypertension and Preeclampsia: Acog Practice Bulletin, Number 222. *Obstet Gynecol* (2020) 135(6):e237-e60. Epub 2020/05/23. doi: 10.1097/AOG.0000000000003891.

6. Khwaja A. Kdigo Clinical Practice Guidelines for Acute Kidney Injury. *Nephron Clin Pract* (2012) 120(4):c179-84. Epub 2012/08/15. doi: 10.1159/000339789.
