# Supplementary material for: Pregnant outcomes of critically ill pregnant patients with pulmonary hypertension: A multicenter retrospective study
Source: Front Cardiovasc Med. 2022 Sep 7;9:872833. doi: 10.3389/fcvm.2022.872833 (PMC9489930; doi:10.3389/fcvm.2022.872833)
Supplement: Supplementary file 2 [file Data_Sheet_2.docx]

Supplementary Material

Table 1. Univariable Logistic Regression of risk factors for MACE in pregnant patients with PH

| Variable | Univariable Logistic regression | |
| --- | --- | --- |
|  | OR (95%CI) | *P* value |
| Age |  |  |
| ≤25y | 2.575 (1.237, 5.361) | 0.011 |
| ≥35y | 0.645 (0.299, 1.391) | 0.263 |
| Nulliparous | 1.302 (0.683, 2.482) | 0.422 |
| BMI≥30 | 0.834 (0.393, 1.769) | 0.636 |
| Saturation≤95% on admission | 1.800 (0.702, 4.618) | 0.221 |
| MAP≤70 mmHg on admission | 1.036 (0.250, 4.294) | 0.961 |
| HCT≤30% on admission | 1.286 (0.526, 3.141) | 0.581 |
| Heart failure on admission | 2.009 (1.065, 3.792) | 0.031 |
| NT-proBNP ≥ 1000 pg/ml on admission | 3.000 (1.337, 6.734) | 0.008 |
| APACHE II score on admission | 1.272 (1.087, 1.490) | 0.003 |
| SOFA score on admission | 1.740 (1.298, 2.334) | 0.000 |
| sPAP |  |  |
| >50 mmHg | 2.495 (1.320, 4.716) | 0.005 |
| >70 mmHg | 3.002 (1.517, 5.942) | 0.002 |
| Combined with Hypertension | 0.674 (0.336, 1.351) | 0.266 |
| Combined with eclampsia/preeclampsia | 0.517 (0.242, 1.106) | 0.089 |
| Combined with AKI | 18.980 (2.314, 155.688) | 0.006 |
| iPH | 1.042 (0.467, 2.322) | 0.920 |
| CHD-PH | 1.217 (0.653, 2.270) | 0.536 |
| Eisenmanger syndrome | 1.254 (0.289, 5.432) | 0.763 |
| LDH-PH | 2.860 (1.156, 7.074) | 0.023 |
| oPH | 0.488 (0.229, 1.040) | 0.063 |
| Delivery weeks ≥32w | 0.958 (0.460, 1.994) | 0.908 |
| Vaginal delivery | 0.414 (0.047, 3.631) | 0.426 |
| Caesarean section | 0.495 (0.243, 1.006) | 0.052 |
| Emergency caesarean section | 2.478 (1.179, 5.208) | 0.017 |
| General anaesthesia | 1.658 (0.838, 3.280) | 0.147 |
| Epidural anaesthesia | 0.742 (0.254, 2.170) | 0.585 |
| Epidural+Spinal anaesthesia | 0.747 (0.396, 1.411) | 0.369 |
| APACHE II score on ICU admission | 1.322 (1.155, 1.513) | <0.001 |
| SOFA score on ICU admission | 1.770 (1.380, 2.270) | <0.001 |
| lactate level on ICU admission | 1.939 (1.250, 3.006) | 0.003 |
| cTnI level on ICU admission | 1.040 (1.015, 1.066) | 0.002 |
| PaO_2_/FiO_2_ < 300 on ICU admission | 2.198 (1.061, 4.555) | 0.034 |
| Inotropic agents | 7.532 (2.312, 24.536) | 0.001 |
| Vasoconstrictors | 7.977 (2.737, 23.253) | <0.001 |
| Anticoagulation | 1.146 (0.592, 2.218) | 0.686 |
| Positive fluid balance on 1^st^ postpartum day | 2.184 (0.597, 7.996) | 0.238 |
| Positive fluid balance on 1^st^-3^rd^ postpartum day | 1.417 (0.215, 9.333) | 0.717 |

AKI: acute kidney injury; APACHE II score: Acute Physiology, Age and Chronic Health Evaluation II score; BMI: body mass index; CHD-PH: PH associated with congenital heart disease; cTnI: cardiac troponin I; HCT: haematocrit; ICU: intensive care unit; iPH: idiopathic PH; LHD-PH: PH associated with left heart disease; MAP: mean arterial pressure; NT-proBNP: N-terminal pro-B-type natriuretic peptide; oPH: PH caused by other diseases; PaO2/FiO2: the ratio of arterial oxygen partial pressure to fractional inspired oxygen; SOFA score: sequential organ failure assessment score; sPAP: systolic pulmonary arterial pressure.

Table 2. Univariable Logistic Regression of risk factors for FACE in pregnant patients with PH

| Variable | Univariable Logistic regression | |
| --- | --- | --- |
|  | OR (95%CI) | *P* value |
| Age |  |  |
| ≤25y | 1.320 (0.604, 2.881) | 0.486 |
| ≥35y | 1.231 (0.588, 2.577) | 0.582 |
| Nulliparous | 0.872 (0.460, 1.651) | 0.673 |
| BMI≥30 | 3.441 (1.415, 8.365) | 0.006 |
| Saturation≤95% on admission | 5.257 (1.728, 23.462) | 0.030 |
| MAP≤70 mmHg on admission | (, ) |  |
| HCT≤30% on admission | 1.050 (0.422, 2.611) | 0.917 |
| Heart failure on admission | 0.643 (0.343, 1.206) | 0.169 |
| NT-proBNP ≥ 1000 pg/ml on admission | 3.707 (1.388, 9.894) | 0.009 |
| APACHE II score on admission | 1.076 (0.932, 1.243) | 0.316 |
| SOFA score on admission | 1.168 (0.917, 1.488) | 0.208 |
| sPAP |  |  |
| >50 mmHg | 0.816 (0.439, 1.515) | 0.519 |
| >70 mmHg | 1.212 (0.599, 2.454) | 0.592 |
| Combined with hypertension | 5.324 (2.236, 12.675) | < 0.001 |
| Combined with eclampsia/preeclampsia | 8.666 (2.945, 25.501) | < 0.001 |
| Combined with AKI | 1.018 (0.246, 4.216) | 0.981 |
| iPH | 1.444 (0.625, 3.336) | 0.389 |
| CHD-PH | 0.447 (0.239, 0.838) | 0.012 |
| Eisenmanger syndrome | 1.553 (0.304, 7.931) | 0.597 |
| LDH-PH | 0.702 (0.282, 1.748) | 0.447 |
| oPH | 2.189 (1.027, 4.662) | 0.042 |
| Delivery weeks ≥32w | 0.032 (0.004, 0.239) | 0.001 |
| Vaginal delivery | 1.018 (0.181, 5.720) | 0.984 |
| Caesarean section | 1.071 (0.520, 2.206) | 0.851 |
| Emergency caesarean section | 0.923 (0.432, 1.975) | 0.837 |
| General anaesthesia | 2.172 (1.018, 4.633) | 0.045 |
| Epidural anaesthesia | 0.662 (0.251, 1.745) | 0.404 |
| Epidural+Spinal anaesthesia | 0.623 (0.326, 1.189) | 0.151 |
| APACHE II score on ICU admission | 1.049 (0.958, 1.148) | 0.302 |
| SOFA score on ICU admission | 1.236 (1.003, 1.524) | 0.047 |
| lactate level on ICU admission | 1.253 (0.898, 1.748) | 0.185 |
| cTnI level on ICU admission | 1.016 (0.994, 1.039) | 0.161 |
| PaO2/FiO2 < 300 on ICU admission | 2.500 (1.276, 4.899) | 0.008 |

AKI: acute kidney injury; APACHE II score: Acute Physiology, Age and Chronic Health Evaluation II score; BMI: body mass index; CHD-PH: PH associated with congenital heart disease; HCT: haematocrit; ICU: intensive care unit; cTnI: cardiac troponin I; iPH: idiopathic PH; LHD-PH: PH associated with left heart disease; MAP: mean arterial pressure; NT-proBNP: N-terminal pro-B-type natriuretic peptide; oPH: PH caused by other diseases; PaO2/FiO2: the ratio of arterial oxygen partial pressure to fractional inspired oxygen; SOFA score: sequential organ failure assessment score; sPAP: systolic pulmonary arterial pressure.

Table 3. Univariable Cox Regression of risk factors for maternal mortality in pregnant patients with PH

| Variable | Univariable Cox regression | |
| --- | --- | --- |
|  | HR (95%CI) | *P* value |
| Age | 0.907 (0.784-0.979) | 0.033 |
| Nulliparous | 4.042 (1.193-13.693) | 0.025 |
| BMI≥30 | 0.901 (0.791-1.026) | 0.116 |
| Saturation≤95% on admission | 0.876 (0.784-0.979) | 0.020 |
| Heart failure on admission | 21.254 (2.714-166.419) | 0.004 |
| NT-proBNP on admission | 1.006 (0.998-1.015) | 0.128 |
| APACHE II score on admission | 1.062 (0.913-1.235) | 0.433 |
| SOFA score on admission | 1.367 (1.072-1.742) | 0.012 |
| sPAP | 1.056 (1.032-1.081) | <0.001 |
| 50-70 mmHg | 1.069 (0.975-1.172) | 0.054 |
| >70 mmHg | 1.316 (1.126-1.538) | <0.001 |
| Combined with Hypertension | 2.842 (0.614-13.146) | 0.181 |
| Delivery weeks | 0.981 (0.890-1.080) | 0.691 |
| Delivery weeks ≥32w | 0.508 (0.109-2.365) | 0.388 |
| Vaginal delivery | 0.184 (0.032-1.050) | 0.057 |
| Caesarean section | 6.840 (2.155-21.708) | 0.001 |
| Emergency caesarean section | 4.500(1.468-13.790) | 0.008 |
| General anaesthesia | 0.352 (0.117-1.060) | 0.063 |
| Epidural anaesthesia | 1.550 (0.191-12.552) | 0.681 |
| Epidural+Spinal anaesthesia | 4.000 (1.205, 13.283) | 0.024 |
| APACHE II score on ICU admission | 1.277 (1.123-1.451) | <0.001 |
| SOFA score on ICU admission | 1.570 (1.244-1.983) | <0.001 |
| lactate level on ICU admission | 2.906 (1.607-5.253) | <0.001 |
| cTnI level on ICU admission | 1.029 (1.009-1.049) | 0.005 |
| PaO2/FiO2 < 300 on ICU admission | 8.813 (1.9.9-40.796) | 0.005 |
| AKI | 0.304 (0.058-1.593) | 0.159 |
| Inotropic agents | 0.037 (0.010-0.132) | <0.001 |
| Vasoconstrictors | 0.004 (0.000-0.033) | <0.001 |
| Anticoagulation | 2.738 (0.906-8.272) | 0.074 |

AKI: acute kidney injury; APACHE II score: Acute Physiology, Age and Chronic Health Evaluation II score; BMI: body mass index; cTnI: cardiac troponin I; ICU: intensive care unit.; NT-proBNP: N-terminal pro-B-type natriuretic peptide; PaO2/FiO2: the ratio of arterial oxygen partial pressure to fractional inspired oxygen; SOFA score: sequential organ failure assessment score; sPAP: systolic pulmonary arterial pressure.
