# Supplementary material for: Pregnant outcomes of critically ill pregnant patients with pulmonary hypertension: A multicenter retrospective study
Source: Front Cardiovasc Med. 2022 Sep 7;9:872833. doi: 10.3389/fcvm.2022.872833 (PMC9489930; doi:10.3389/fcvm.2022.872833)
Supplement: Supplementary file 3 [file Data_Sheet_3.docx]

Supplementary Material


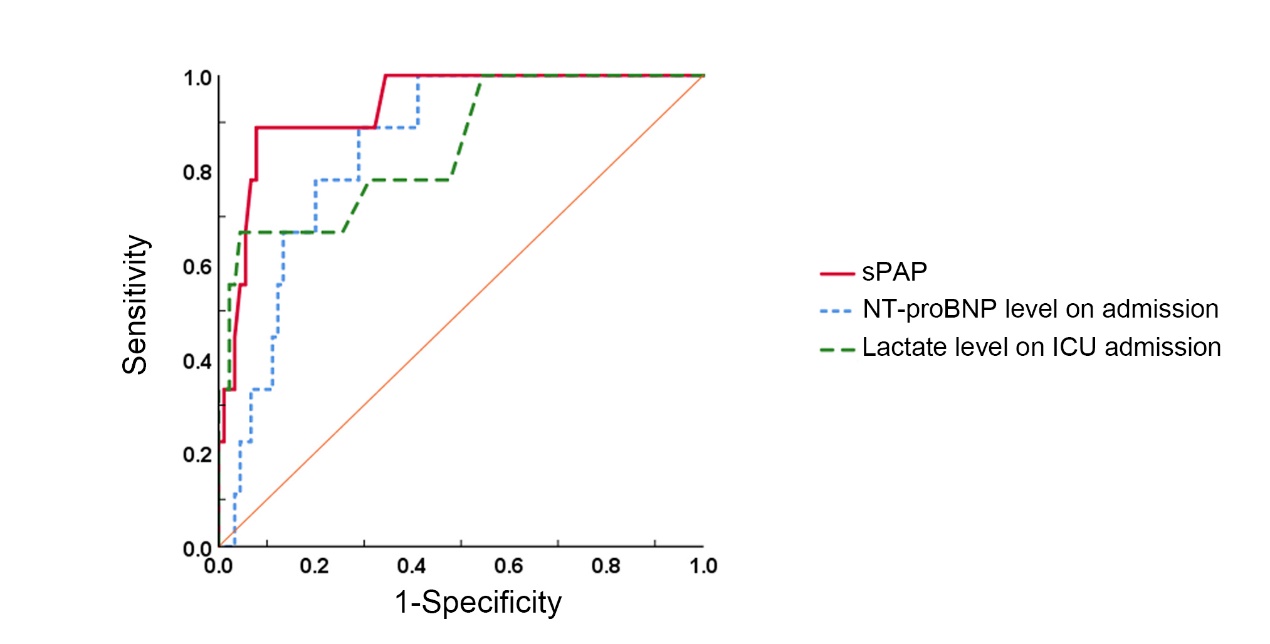


|  | Cut-off value | *P* | AUC (95% CI) | Sensitivity | Specificity |
| --- | --- | --- | --- | --- | --- |
| sPAP | 70 | < 0.001 | 0.932 (0.861, 1000) | 0.889 | 0.711 |
| NT-proBNP level on admission | 1000 | 0.001 | 0.843 (0.748, 0.938) | 0.889 | 0.778 |
| Lactate level on ICU admission | 2.85 | 0.001 | 0.846 (0.704, 0.987) | 0.667 | 0.956 |

Figure.1 Receiver operative characteristic (ROC) curve for using sPAP, NT-proBNP level on admission, lactate level on ICU admission to predict in-hospital death. ICU: intensive care unit.; NT-proBNP: N-terminal pro-B-type natriuretic peptide; sPAP: systolic pulmonary arterial pressure.
